# Reconciling Phylodynamics with Epidemiology: The Case of Dengue Virus in Southern Vietnam

**DOI:** 10.1093/molbev/mst203

**Published:** 2013-10-22

**Authors:** David A. Rasmussen, Maciej F. Boni, Katia Koelle

**Affiliations:** ^1^Biology Department, Duke University; ^2^Oxford University Clinical Research Unit, Wellcome Trust Major Overseas Programme, Ho Chi Minh City, Vietnam; ^3^Centre for Tropical Medicine, Nuffield Department of Clinical Medicine, University of Oxford, Oxford, United Kingdom; ^4^Fogarty International Center, National Institutes of Health, Bethesda, MD

**Keywords:** phylodynamics, coalescent, demographic inference, infectious diseases

## Abstract

Coalescent methods are widely used to infer the demographic history of populations from gene genealogies. These approaches—often referred to as phylodynamic methods—have proven especially useful for reconstructing the dynamics of rapidly evolving viral pathogens. Yet, population dynamics inferred from viral genealogies often differ widely from those observed from other sources of epidemiological data, such as hospitalization records. We demonstrate how a modeling framework that allows for the direct fitting of mechanistic epidemiological models to genealogies can be used to test different hypotheses about what ecological factors cause phylodynamic inferences to differ from observed dynamics. We use this framework to test different hypotheses about why dengue serotype 1 (DENV-1) population dynamics in southern Vietnam inferred using existing phylodynamic methods differ from hospitalization data. Specifically, we consider how factors such as seasonality, vector dynamics, and spatial structure can affect inferences drawn from genealogies. The coalescent models we derive to take into account vector dynamics and spatial structure reveal that these ecological complexities can substantially affect coalescent rates among lineages. We show that incorporating these additional ecological complexities into coalescent models can also greatly improve estimates of historical population dynamics and lead to new insights into the factors shaping viral genealogies.



## Introduction

The field of phylodynamics is concerned with how various ecological and evolutionary processes act or interact to shape genealogies and patterns of genetic diversity (Grenfell et al. 2004; Volz et al. 2013). A major focus of phylodynamics has also been on what can be considered the inverse problem—given a genealogy, can the processes that generated the genealogy be inferred? With respect to this question, most effort has been focused on inferring the demographic history of populations from genealogies using coalescent-based methods such as the popular Bayesian Skyline approach (Strimmer and Pybus 2001; Drummond et al. 2005). These methods have become especially popular among epidemiologists studying the population dynamics of infectious diseases, particularly rapidly evolving RNA viruses like influenza, dengue, hepatitis C, and HIV (Pybus et al. 2001; Rambaut et al. 2008; Gray et al. 2009; Bennett et al. 2010).

Infectious diseases also present an opportunity to test phylodynamic methods in situations where epidemiological data like time series of case reports are available alongside sequence data, allowing phylodynamic reconstructions of population dynamics to be compared against patterns observed through hospital- or community-reported incidence. Reassuringly, in many cases, phylodynamic estimates have been in line with observed disease dynamics. A very striking example of such congruence was provided by Rambaut et al. (2008), who reconstructed seasonal influenza A dynamics consistent with the strongly annual fluctuations observed in surveillance data. Phylodynamic methods have also been used to successfully reconstruct the early, exponential growth phase of emerging epidemics (Pybus et al. 2001; Lemey et al. 2003; Dearlove and Wilson 2013). Yet, in other cases, phylodynamic estimates have differed widely from observed or expected disease dynamics. This has often been the case with pathogens undergoing complex seasonal or multiannual dynamics (Amore et al. 2010; Bennett et al. 2010; Siebenga et al. 2010; Lin et al. 2013). Although the inability to capture fluctuations in population size at fine temporal resolution can partially be attributed to insufficiently dense sampling, cases have even been found where dynamics inferred from genealogies are out of phase with case report data (Bennett et al. 2010).

Discrepancies between phylodynamic estimates and observed dynamics highlight some of the technical issues that need to be addressed if phylodynamic methods are to become a reliable tool in epidemiology and other fields. One major concern is whether the coalescent models often used in phylodynamic inference are appropriate for populations undergoing complex population dynamics, as is often the case for infectious diseases. This is important for inference because it is the coalescent model that provides the probabilistic framework necessary to compute the likelihood of a particular demographic model given a genealogy. Coalescent models commonly used in traditional population genetics assume that the coalescent rate is inversely proportional to the effective population size *N*_e_. For infectious diseases, changing transmission rates can also affect coalescent rates (Volz et al. 2009; Frost and Volz 2010). Therefore, the dynamics of *N*_e_ inferred from genealogies using standard coalescent models need to be interpreted carefully for pathogens as they may not reflect the true underlying disease dynamics. Additional ecological complexities can also seriously bias estimates if not properly taken into account. For example, different forms of population structure can bias estimates obtained using coalescent models that do not take into account the possibility of different lineages being in different populations (Carrington et al. 2005; Pybus and Rambaut 2009; Heller et al. 2013). These issues make it difficult to assess whether inferences drawn from phylodynamic analyses are reliable or are, at least in part, artifacts of the coalescent models used for inference.

To explore some of these issues, we used dengue virus as a case study in phylodynamic inference. Dengue is a mosquito-borne flavivirus and has been the subject of several previous phylodynamic studies, which have had various degrees of success reconstructing dengue’s complex epidemiological dynamics (Schreiber et al. 2009; Bennett et al. 2010; McElroy et al. 2011; Raghwani et al. 2011). In this article, we limit our attention to dengue serotype 1 (DENV-1) in southern Vietnam, for which a large number of sequence samples and reliable hospitalization data are both available.

We were also interested in DENV-1 because, as shown below, we were unable to reconstruct the highly seasonal incidence patterns observed in hospitalization data using Bayesian Skyline methods. Although there are many plausible explanations for this discrepancy, we explored three factors particularly relevant to dengue. These were as follows: 1) Dengue’s seasonality and nonlinear transmission dynamics, which lead to rapid fluctuations in dengue incidence; 2) vector-borne transmission and the population dynamics of mosquitoes; and 3) spatial structure in the host population arising from the spatial heterogeneity of southern Vietnam. Although all three of these factors play a crucial role in dengue’s ecological dynamics, it is less clear how each factor acts to shape viral genealogies and therefore affects inferences drawn using coalescent-based methods.

To understand how each of these factors affects phylodynamic estimates drawn from the DENV-1 genealogy, we used a mechanistic modeling framework that allowed us to formulate each of the three proposed factors as a simple compartmental epidemiological model: a seasonal susceptible-infected-recovered (SIR) model, a vector-borne SIR model, and a spatially structured SIR model. We then derived coalescent models corresponding to each of the epidemiological models using the framework presented in Volz et al. (2009) and Volz (2012). With these coalescent models, we were able to directly fit each of the epidemiological models to the DENV-1 genealogy and explore how each factor affects the coalescent process. By comparing the relative fit of each model to the genealogy, we were able to gain insight into which factors are most important in shaping the DENV-1 genealogy. Moreover, the best-fitting epidemiological models did much better than standard coalescent models in reconstructing population dynamics consistent with the dengue hospitalization data, showing that incorporating mechanistic modeling approaches into phylodynamic inference can greatly improve estimates of historical population dynamics.

## Results

**Fig. 1.**
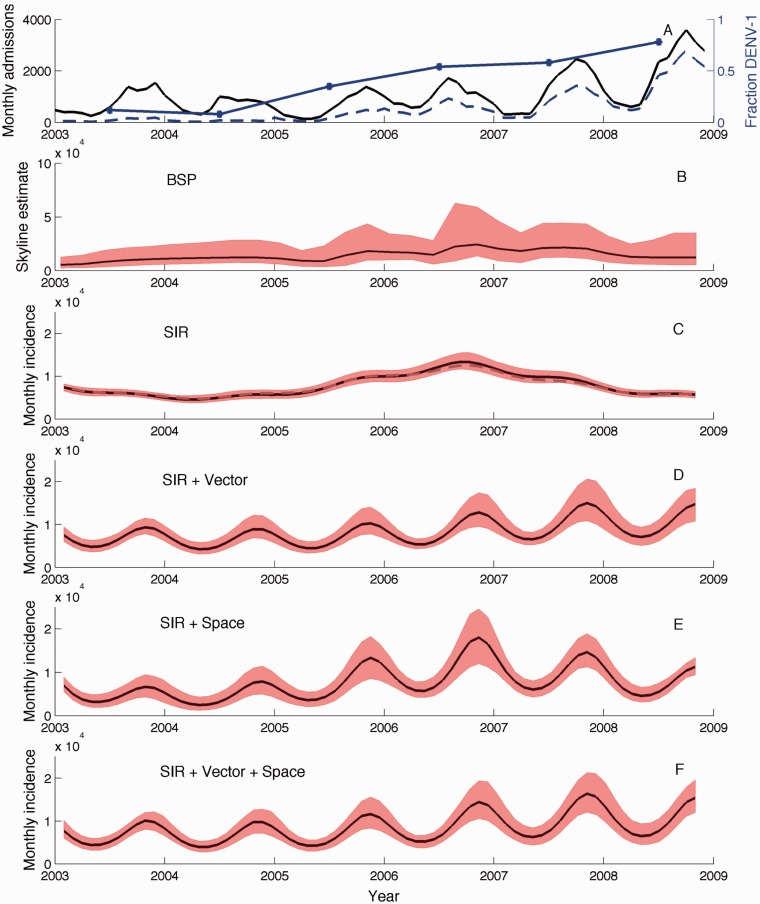
Population dynamics of dengue in southern Vietnam. (*A*) Absolute number of dengue hospital admissions each month in HCMC (black), yearly relative abundance of DENV-1 among RT-PCR positive cases (blue), and the extrapolated number of DENV-1 hospitalizations (dashed blue). (*B*) BSP inferred from the DENV-1 sequences. Black lines show the median posterior estimates and shaded red regions give the 95% credible intervals. (*C*) Incidence inferred under the seasonal SIR model from the DENV-1 genealogy. Incidence estimates are reported as the absolute number of cases occurring each month. The dashed gray line shows the median estimate obtained from the HCMC-specific genealogy. (*D*) Incidence inferred under the vector-borne model. (*E*) Incidence inferred under the spatially structured model in HCMC. (*F*) Incidence inferred under the combined model with vectors and spatial structure.

Dengue is hyperendemic in southern Vietnam with all four serotypes commonly circulating together. Previous epidemiological studies have shown that incidence is consistently high in the region with an annual attack rate in children estimated to be approximately 10% (Thai et al. 2005, 2011). Case reports collected at hospitals in Ho Chi Minh City (HCMC) between 2003 and 2008 indicate that transmission can occur year-round, although incidence is highly seasonal with a strong annual periodicity (fig. 1*A*; Materials and Methods).

The hospitalization data shown in figure 1*A* include all four serotypes but may not be representative of any particular serotype. We therefore used viral isolates serotyped using reverse transcriptase-polymerase chain reaction (RT-PCR) to determine the fraction of isolates belonging to each of the four dengue serotypes over time (Materials and Methods). As shown in figure 1*A*, the proportion of DENV-1 isolates dramatically increased from around 2004 onward. This trend is consistent with regional level data that indicate DENV-1 replaced DENV-2 as the dominant serotype in southern Vietnam while the relative abundances of DENV-3 and DENV-4 remained low over this period of time (Vu et al. 2010). Because of the predominance of DENV-1 over the time period studied, we focused on this serotype in our phylodynamic analysis, using the fraction of DENV-1 viral isolates to estimate monthly DENV-1 incidence from the hospitalization data (fig. 1*A*). Although the hospitalization data are likely representative of DENV-1 dynamics, the total incidence of DENV-1 is likely much higher because only a small fraction of dengue cases result in hospitalization.

To determine whether we could reconstruct the dynamics observed in the dengue hospitalization data from sequence data, we inferred the genealogy of 237 DENV-1 whole genome sequence samples collected between 2003 and 2008 from dengue patients living throughout southern Vietnam (Materials and Methods). The maximum clade credibility (MCC) genealogy for these samples is shown in supplementary figure S1, Supplementary Material online. Figure 1*B* shows the population dynamics inferred, along with the genealogy, using BEAST in the form of a Bayesian Skyline Plot (BSP). Although we do recover the increase in DENV-1 that occurred starting around 2004, other aspects of the dynamics observed in the hospitalization data are absent in the BSP. Most noticeably, the small fluctuations of DENV-1 inferred from the genealogy do not seem consistent with the large seasonal fluctuations in the hospitalization data (fig. 1*A* and *B*). Although in theory this could be due to inadequate sampling, exploratory simulations using sequence data simulated under dengue-like dynamics showed that the large seasonal fluctuations should be recoverable given the current sample size (simulations not shown). Aside from the discrepancy in seasonal dynamics, the BSP also shows DENV-1 incidence peaking in 2006 and then declining whereas the hospitalization data shows the peak in seasonal incidence increasing each year from 2004 to 2008.

Because the sequence samples were collected from patients living within a large geographic region, we also tried to reconstruct DENV-1 dynamics only within HCMC, reasoning that it may be easier to reconstruct seasonal dynamics on a more limited spatial scale than all of southern Vietnam. To do so, we performed a second Bayesian Skyline analysis with a genealogy from which all non-HCMC samples were removed. However, the Bayesian Skyline reconstruction of dynamics within HCMC also failed to recover the large seasonal fluctuations in DENV-1 incidence (supplementary fig. S2, Supplementary Material online).

### Seasonality

Given the large discrepancy in seasonal dengue dynamics between the BSP and the hospitalization data, we first considered whether an epidemiological model that explicitly considered seasonality and nonlinear transmission dynamics might outperform the BSP. We therefore fit a SIR model with seasonal forcing to the DENV-1 genealogy using a coalescent model derived from the SIR model (Materials and Methods).

**Fig. 2.**
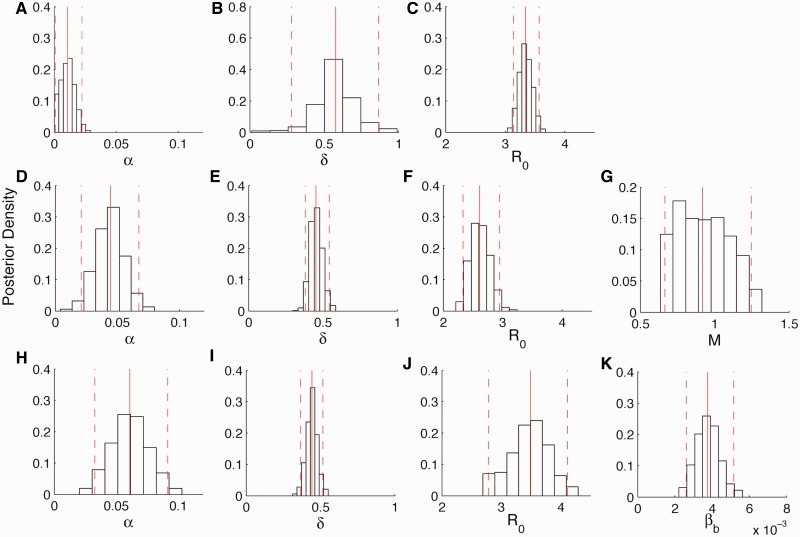
Posterior densities of the parameters inferred from the DENV-1 genealogy. Solid red lines indicate the median and dashed red lines indicate the 95% credible intervals of the posterior densities. The parameter *α* is the seasonal amplitude, *δ* is seasonal phase parameter, *R*_0_ is the basic reproduction number, *M* is the ratio of mosquito to human population sizes in the vector-borne model, and 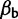 is the transmission rate between populations in the spatially structured model. (*A–C*) Estimates for the seasonal SIR model, (*D–G*) estimates for the vector-borne model, and (*H–K*) estimates for the spatially structured model.

The population dynamics inferred from the DENV-1 MCC genealogy under the seasonal SIR model were qualitatively very similar to the dynamics in the BSP, with the seasonal fluctuations in incidence still an order of magnitude lower than those observed in the hospitalization data (fig. 1*C*). Coinciding with the small fluctuations in incidence, epidemiological parameters estimated directly from the genealogy also indicated very low seasonal amplitude (quantifying the strength of seasonality) and a difficulty in identifying the seasonal phase (fig. 2*A* and *B*). Incidence estimated from the genealogy is also much higher than the number of hospital admissions, which we expected based on the fact that most dengue cases are not severe enough to require hospitalization. The basic reproduction number *R*_0_ was estimated to be slightly higher than three (fig. 2*C*), consistent with the range of serotype-specific *R*_0_ values previously reported for dengue in southeast Asia (Ferguson et al. 1999; Thai et al. 2005). As in the BSP, the inferred dynamics show DENV-1 incidence peaking in 2006 and then steadily declining, at odds with the continued growth in peak incidence each season observed in the hospitalization data. Similar dynamics were inferred from the genealogy containing only samples from HCMC (fig. 1*C*).

To explore how uncertainty in the genealogy, especially with respect to the coalescent times, might affect our estimates, we additionally fit the seasonal SIR model to ten random trees sampled from the BEAST posterior tree distribution. Reconstructed dynamics did not significantly differ between trees, suggesting estimates were largely robust to phylogenetic uncertainty (supplementary fig. 3*A*, Supplementary Material online). The seasonal SIR model therefore appears unable to reconstruct dynamics consistent with the hospitalization data regardless of the geographic distribution of samples or the particular genealogy used for inference.

### Vector Dynamics

Dengue is a vector-borne virus spread by *Aedes* mosquitoes and the seasonality in dengue transmission presumably arises from fluctuations in mosquito population densities. Yet, the seasonal SIR model fit above does not explicitly consider vector-borne transmission or mosquito population dynamics. To see whether ignoring the vector population in the coalescent model could be distorting population dynamic inferences drawn from the genealogy, we fit a mechanistic vector-borne SIR model with seasonality in mosquito birth rates to the DENV-1 genealogy (Materials and Methods).

The population dynamics inferred from the DENV-1 genealogy under the vector-borne model show much larger seasonal fluctuations in incidence and correspond to the hospitalization data much better than those inferred under the directly transmitted model (fig. 1*D*). These pronounced seasonal fluctuations arise from an estimated amplitude parameter that is much higher under the vector-borne model than the directly transmitted SIR model (fig. 2*D*). We were also able to reconstruct the sustained growth in peak DENV-1 incidence each season through 2008, which we were unable to capture using the BSP or the directly transmitted model. Overall, a model comparison using Bayes factors showed that the vector-borne model provided a much better fit to the DENV-1 genealogy, with the posterior odds highly favoring the vector-borne model over the directly transmitted model (table 1).

**Table 1.** Comparison of the Models Fit to the DENV-1 Genealogy.

Model	Median Posterior^a^	Bayes Factor^b^
Seasonal SIR	−2,342.4	—
SIR + vector	−2,271.1	71.0
SIR + space	−2,253.9	88.3
SIR + vector + space	−2,247.9	93.7

^a^Median log posterior probability.

^b^Log Bayes factor.

We were also able to obtain much more precise estimates of the seasonal phase parameter using the vector-borne model (fig. 2*E*). The estimated phase coincides with a peak in mosquito population densities occurring in May or June, the same time at which *Aedes aegypti* densities peak in independent data from the Pasteur Institute in HCMC (Coudeville and Garnett 2012). *R*_0_ under the vector-borne model was estimated to be slightly lower than three (fig. 2*F*), again consistent with the range of *R*_0_ estimates in the literature (Ferguson et al. 1999; Thai et al. 2005). We were also able to obtain an estimate of the seasonal average of *M*, the ratio of mosquito to human population sizes, at a value close to one (fig. 2*G*). For comparison, estimates from other areas of the world have reported the number of *A. aegypti* per person to range from 0.2 to over 60.0, although most reported values fall below one (Focks and Chadee 1997; Morrison et al. 2004; Koenraadt et al. 2008; Jeffery et al. 2009).

To gain intuition about why the vector-borne model was able to capture the population dynamics of DENV-1 better than the directly transmitted model, we studied the coalescent process for a vector-borne pathogen in the supplementary appendix, Supplementary Material online. Our mathematical analysis revealed that a vector-borne pathogen will in general have a lower rate of coalescence than a directly transmitted pathogen, although how much lower depends on the ratio of mosquito to human population sizes, *M*. As *M* increases, so does the number of infected mosquitoes. A larger number of infected mosquitoes decrease the coalescent rate in a way similar to how larger population sizes decrease the coalescent rate in standard population genetics models. Thus, the larger *M* is, the lower the coalescent rate for a vector-borne disease will be relative to directly transmitted pathogen, although the relationship between *M* and the coalescent rate is nonlinear (supplementary fig. S4, Supplementary Material online).

**Fig. 3.**
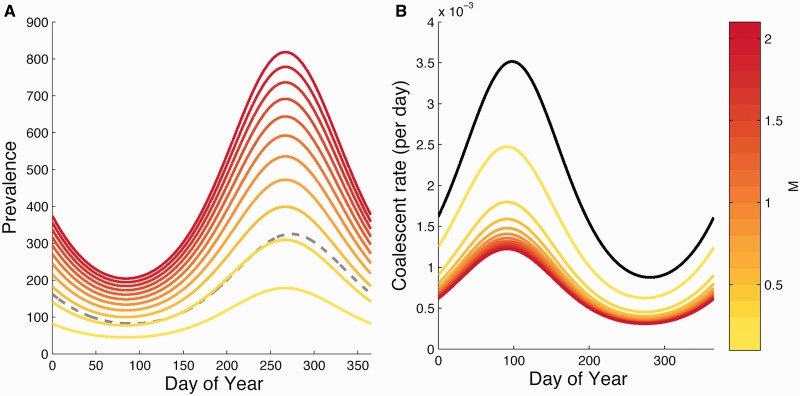
Comparison of seasonal coalescent rates and mosquito population sizes for the direct transmission model and the vector-borne model. (*A*) Simulated seasonal prevalence of the disease in humans and mosquitoes. The different colored lines show disease prevalence in mosquitoes assuming different values of *M*. Prevalence in humans (dashed-gray) is also seasonal, and constrained to be the same for both the direct and vector-borne model by keeping *R*_0_ constant. (*B*) Seasonal coalescent rates for both models. The black line shows the seasonal coalescent rate for the direct transmission model and the different colored lines are the coalescent rates for the vector-borne model.

The seasonal fluctuations in the coalescent rate also become increasingly damped for the vector-borne model relative to the direct transmission model as *M* increases (fig. 3). At high values of *M*, the coalescent rate is low year-round because the number of infected mosquitoes remains large year-round. The damped fluctuations in the coalescent rate will result in coalescent events being more uniformly distributed throughout the year in the genealogy, which will be interpreted as small fluctuations in human incidence under a coalescent model for a directly transmitted pathogen. It is therefore possible for a vector-borne pathogen to induce large seasonal fluctuations in human incidence, but to infer low-amplitude oscillations in human incidence under a coalescent model that ignores the vector population. Interestingly, our estimate of *M* around one falls in a part of parameter space in which this would likely occur. These results therefore explain why the vector-borne model was able to better reconstruct the highly seasonal patterns of human DENV-1 incidence.

We also note that, on average, incidence inferred under the vector-borne model was approximately 10% lower than under the direct transmission model. This makes sense given the lower rate of coalescence for a vector-borne pathogen. Under both coalescent models, there is a certain number of infected humans that maximizes the likelihood of observing a given coalescent event. However, for the vector-borne model, this number of infected humans needs to be lower to increase the coalescent rate to compensate for the effect of the vector. To have fewer infected humans, the basic reproduction number *R*_0_ is estimated to be lower under the vector-borne model (fig. 2*F*). This in turn likely explains why we were able to capture the continued rise in peak DENV-1 incidence each season through 2008 under the vector-borne model while incidence peaked too early under the direct model. The higher *R*_0_ estimated under the direct transmission model causes the susceptible human population to be rapidly depleted and therefore incidence to decline after 2006. In comparison, the lower *R*_0_ estimated under the vector-borne model allows for a more gradual depletion of susceptible humans and therefore a sustained, gradual rise in DENV-1 incidence each season.

### Spatial Structure

There is considerable spatial heterogeneity in dengue transmission dynamics across southern Vietnam, which includes large urban centers like HCMC as well as the less densely populated provinces to the north and west and the rural Mekong Delta region to the south. In our third model, we therefore considered how spatial structure may affect inferences drawn from the DENV-1 genealogy. As a starting point, we considered a spatially structured model with two populations: a HCMC and a non-HCMC population. Although this simple model cannot account for all of the spatial heterogeneity in the region, including a non-HCMC population may allow us to more accurately infer dynamics within HCMC by controlling for the movement of lineages in and out of the city. To fit this structured model, we used the coalescent framework developed in Volz (2012) to compute the probability of each lineage being in either the HCMC or non-HCMC population conditional on the location of the external lineages at the time of sampling (Materials and Methods). Under this model, the coalescent rate between different lineages can differ depending on each lineage’s probability of being in each population. For example, two lineages with high probabilities of being in HCMC will have a higher expected coalescent rate than two lineages with a high probability of being in different populations.

Incidence patterns inferred from the genealogy using the spatially structured model show large seasonal fluctuations in incidence consistent with the hospitalization data (fig. 1*E*). The seasonality parameters and *R*_0_ for the structured model are shown in figure 2*H*–*K*. However, the dynamics inferred under the spatially structured model show the highest seasonal peak in incidence occurring in 2006, with subsequent years having lower peak incidence. We therefore also fit a combined model with both vector-borne transmission and spatial structure. Incidence patterns inferred under the combined model show both large seasonal fluctuations and continued growth in peak incidence each season from 2004 to 2008, consistent with the hospitalization data (fig. 1*F*). Parameter estimates for the combined model are shown in supplementary figure 5, Supplementary Material online. Bayes factor comparisons also showed that while both the vector-borne and spatially structured model fit the genealogy significantly better than the unstructured model, the combined model fits better than either of the two models individually (table 1). The population dynamics reconstructed under the best-fitting combined model also appear robust to phylogenetic uncertainty (supplementary fig. 3*B*, Supplementary Material online).

The spatially structured model we fit above only assumed seasonality in the HCMC population. However, both hospitalization and notifiable disease data (Cuong et al. 2013) indicate that all of Vietnam’s southern provinces experience strong seasonal fluctuations in incidence. These data further indicate that seasonal outbreaks begin and peak 1 to 3 months earlier in the provinces than in HCMC (supplementary fig. 6*A* and *B*, Supplementary Material online). We therefore fit a second model that included seasonality in both the HCMC and non-HCMC populations and allowed the amplitude and phase of seasonality to vary between the two populations. Although this more complex model did not fit the genealogy significantly better (Bayes factor < 1.0), we were able to reconstruct the differences in seasonality between the HCMC and non-HCMC populations observed in hospitalization data (supplementary fig. 6*C*, Supplementary Material online). The reconstructed incidence clearly shows that the dengue season begins in the provinces about 1 to 3 months earlier than in HCMC. Thus, including spatial structure in the coalescent model not only allowed us to improve our estimates of the population dynamics in HCMC, but to detect spatiotemporal differences in dengue transmission across the region.

**Fig. 4.**
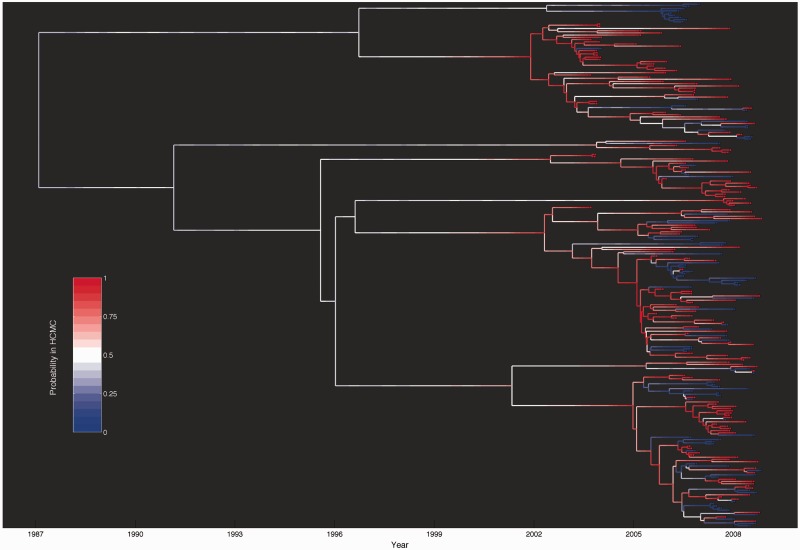
DENV-1 genealogy showing the probability that each lineage is in HCMC. Lineage state probabilities were computed under the spatially structured model using the median posterior values of all parameters. The colored boxes at the tips indicate the population from which the lineage was sampled. Red indicates HCMC and blue indicates the non-HCMC population.

Previous phylogeographic analyses of dengue in southern Vietnam have found evidence for frequent movement of lineages in and out of HCMC (Rabaa et al. 2010; Raghwani et al. 2011). Consistent with these findings, we estimated a relatively high between-population transmission rate (fig. 2*K*). Using this rate along with the other estimated parameters and population dynamics, we computed the probability of each lineage being in HCMC over time (fig. 4). The mapping of lineage state probabilities onto the tree indicated that many different lineages have been imported and exported in and out of HCMC; it is likely that some lineages have even moved in and out of HCMC multiple times since DENV-1 reemerged as the dominant serotype in the early 2000s.

**Fig. 5.**
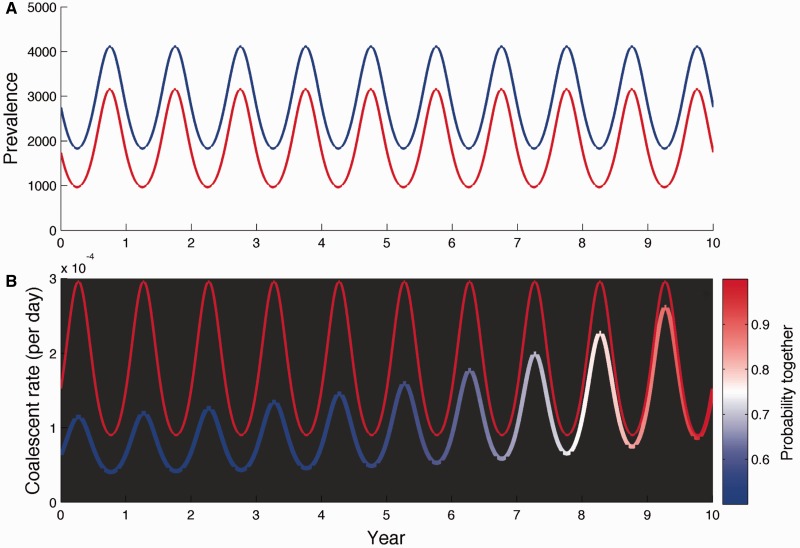
(*A*) Simulated seasonal dynamics for a structured population with a focal (red) and global (blue) population representing the HCMC and non-HCMC populations, respectively. (*B*) Expected coalescent rates for two lineages both sampled in HCMC at the end of year ten. The solid red line shows the strong seasonal fluctuations in coalescent rates in HCMC under an unstructured model. The colored line is the coalescent rate under the spatially structured model where the color shows how the probability of the lineages being together in the same population changes over time.

To better understand how the movement of lineages in a spatially structured population shapes the genealogy, we simulated dengue-like dynamics under the spatial SIR model with parameters close to what we inferred from the DENV-1 genealogy (fig. 5*A*). The expected coalescent rates for two hypothetical lineages sampled in HCMC are shown in figure 5*B*. If we ignore the spatial structure of the population and assume that the two lineages remain in HCMC over time, the rate at which these lineages coalesce fluctuates between high and low as the prevalence cycles between low and high, giving the strong signal of seasonality we expect to see in the timing of coalescent events.

In a spatially structured population, however, our two hypothetical lineages may not remain in the same population indefinitely going into the past because of movement of lineages between populations. This results in a decline in the probability that our two hypothetical lineages remain in the same population as we recede into the past. As this happens, the coalescent rate decreases and the seasonal fluctuations in the coalescent rate also dampen going back in time (fig. 5*B*). In the very recent past, both lineages retain a high probability of being in HCMC and so the coalescent rate reflects the highly seasonal coalescent process in HCMC. However, in the more distant past, the coalescent rate remains low year-round because of the higher probability of the lineages being in different populations. Thus, spatial structure destroys the strong signal of seasonality we expect in the timing of coalescent events in an unstructured population. This likely explains why were able to infer strong seasonality using the structured coalescent approach but were unable to do so simply by removing samples from outside of HCMC from the genealogy. The rapid movement of lineages in and out of HCMC means that many of the lineages sampled in HCMC were not in HCMC in the recent past. Only by taking into account the probable location of lineages through time can we detect the signal of seasonality in the timing of coalescent events within a given population.

## Discussion

Our phylodynamic analysis of DENV-1 shows that, while it is possible to reconstruct complex population dynamics from genealogies, additional ecological factors may need to be included in coalescent models in order for demographic inferences to be accurate. For DENV-1, we were unable to detect the large seasonal fluctuations in dengue incidence using the popular Bayesian Skyline method or even a coalescent model derived from a SIR epidemiological model that allowed for seasonality. However, using models that included either vector population dynamics or spatial structure in the host population, we were able to successfully reconstruct DENV-1 dynamics. The substantially better fit of these two more complex models indicates that vector dynamics and spatial heterogeneity likely play a large role in shaping the genealogy of dengue.

More generally, our results add to the mounting body of evidence that both population dynamics and structure can strongly impact the shape of viral genealogies (Frost and Volz 2010; Bahl et al. 2011; Pybus et al. 2012; Duke-Sylvester et al. 2013; Robinson et al. 2013; Stadler and Bonhoeffer 2013). When conducting phylodynamic analyses, this dependence of phylogeny on ecology can be both good and bad. On the upside, the strong influence of ecological factors means that genealogies contain valuable information about the dynamics and structure of populations that may be absent in other sources of data. For example, we were able to infer the transmission rate of DENV-1 between HCMC and the non-HCMC population, about which hospitalization records contain no information. On the downside, our results for DENV-1 suggest that we may need to include ecological factors in coalescent models that may not be of primary interest to us or we know little about when inferring population dynamics from genealogies.

Although it is difficult to know a priori what ecological factors need to be included in a coalescent model for a particular pathogen, different factors can be tested by formulating them in terms of a mechanistic model that can be fit to genealogies through the appropriate coalescent model. These models do not need to be very complex—our vector-borne model and spatial model were both very simple. The appropriate coalescent model can then be derived from the forward-time model using the coalescent framework of Volz (2012). Different models can then be compared using model selection, as we did using Bayes factors. The advantage of this approach is that mechanistic insights into the factors shaping genealogies can be found, increasing our general knowledge about which factors are most important in shaping the genealogies of different pathogens.

For dengue, it is interesting to consider why vector-borne transmission and spatial structure had such a large impact on our estimates. Although mosquitoes play an integral role in dengue’s ecological dynamics, it is not clear from standard coalescent theory why the vector population needs to be considered. The pairwise coalescent rate under most standard population genetics models depends only on the population size. If we assume that the population size for an infectious pathogen is equivalent to the number of infected hosts, we might think that the coalescent rate should show large fluctuations as the number of infected humans rises and falls. However, the coalescent model derived from the vector-borne SIR model tells us that it is not only prevalence in the human population that is important, but that mosquito population densities are also important. As the mosquito population increases so too does the number of infected mosquitoes, resulting in a lower probability that a given lineage in a human will coalesce with a given lineage in a mosquito. If mosquito population densities are high year-round, the coalescent rate will remain low year-round even if there are large fluctuations in human infections. Thus, unless the coalescent model includes the vector, estimates of the strength of seasonality will be biased.

Given the large amount of spatial heterogeneity in dengue dynamics in southern Vietnam and the widespread movement of people in the region (Rabaa et al. 2010; Raghwani et al. 2011), it does not seem too surprising that including spatial structure in the coalescent model improved our ability to reconstruct population dynamics in a particular population like HCMC. In highly structured populations, lineages in different isolated communities may have little probability of coalescing with one another, especially if transmission between those communities is rare. In this case, the distribution of coalescent events over the genealogy will depend more on the spatial structure of the population than on the dynamics within any particular community. This is one of the reasons why phylodynamic estimates of population sizes in spatially structured populations are usually taken to be a measure of the relative genetic diversity of the population, which may not reflect the true population dynamics (Carrington et al. 2005; Pybus and Rambaut 2009). However, in many cases we may not be interested in patterns of relative genetic diversity but actually want to reconstruct the population dynamics within a particular focal population. As we showed for HCMC, it is possible to reconstruct the dynamics in a focal population by taking into account the movement of lineages and their probable locations through time in the coalescent model. Remarkably, including spatial structure in the coalescent model even allowed us to detect the short lag in time between the beginning of the dengue season in the provinces and the beginning of the seasonal outbreak in HCMC.

Our experience with DENV-1 may also shed some light on why phylodynamic estimates of seasonal population dynamics have been successful for some pathogen populations but not others. Annual seasonal dynamics have been inferred from viral sequence data before, most notably for influenza A in temperate regions (Rambaut et al. 2008). However, in the case of influenza, there is no year-round transmission in temperate regions and the viral population is seeded by imported viruses each year (Nelson et al. 2007; Bedford et al. 2010). Therefore, looking back in time, all lineages sampled during a given season that descended from one of the imported lineages will coalesce at the beginning of the season. Because of this, influenza genealogies contain a strong signature of exponential growth each year—seasonality is so strong that it masks the effects of global population structure on the genealogy. However, if prevalence varies seasonally but the pathogen can still persist in the focal population year-around, accounting for population structure might be necessary. In less seasonal populations, some lineages may remain in the focal population for many seasons going into the past while other lineages may have left the focal population, obscuring the local population dynamics in the genealogy. This may account for why previous phylodynamic studies of populations with seasonal dynamics but year-round persistence were unable to reconstruct accurate seasonal fluctuations in prevalence from genealogies (Amore et al. 2010; Bennett et al. 2010). In such cases, it would be interesting to see if our strategy of subdividing the population into a global and a focal population in the coalescent model would improve estimates of seasonality.

Adding ecological realism to our coalescent models greatly improved our ability to accurately infer DENV-1 dynamics, but do our estimates of dengue incidence accurately reflect the true number of dengue infections? If they do, it would be of great significance to dengue epidemiology, as determining overall disease burden remains challenging because clinical cases represent only a small fraction of all cases. However, we are somewhat skeptical that our estimates accurately reflect the true incidence of dengue because there are several ecological factors that we did not consider in our models that could bias our estimates. For one, our models assume that there is no heterogeneity in transmission rates, whereas in reality there is likely a large amount of variation in the rate at which different mosquitoes bite and the rate at which different humans are bitten (Scott and Morrison 2010). Variation in transmission rates will increase coalescent rates, akin to how reproductive variance reduces effective population size and increases coalescent rates in standard population genetics models (Griffiths and Tavare 1994; Pybus et al. 2001; Charlesworth 2009; Koelle and Rasmussen 2012). Transmission heterogeneity would therefore cause us to underestimate the true number of infections from the genealogy. One in theory could use the ratio of the observed number of infections to the estimated effective population size to infer the extent of transmission heterogeneity, as was done by Magiorkinis et al. (2013), but again for dengue we have no way of knowing the true number of infections. We also did not consider fine-scale spatial structure within HCMC and the provinces. In contrast to the effect of transmission variance, unaccounted for spatial heterogeneity would decrease the rate of coalescence, similar to an increase in the effective population size in standard population genetics models (Wright 1943; Beerli and Felsenstein 2001; Laporte and Charlesworth 2002). Thus, if there is strong local spatial structure, our estimates of incidence will be biased upwards. It is possible that local population structure counteracts the affects of variable transmission rates so that these two sources of potential bias cancel each other out, but the relative magnitude of each is unknown. We therefore urge caution in interpreting our estimates as representative of the true number of dengue cases.

There are certainly many other ecological factors that could distort inferences from genealogies that we did not consider for DENV-1. Notably, we did not consider interactions between DENV-1 and the remaining three dengue serotypes, or interactions between different DENV-1 genotypes. However, there was only a single dominant DENV-1 genotype circulating in the population and single-serotype SIR models were sufficient to capture the rise in DENV-1 incidence that occurred in Vietnam during the period we considered. However, we cannot rule out selection acting within this genotype. Theoretical work has shown that both purifying and directional selection increases coalescent rates deeper in the genealogy, similar to a decrease in the past effective population size (O’Fallon et al. 2010; Walczak et al. 2012; Neher and Hallatschek 2013). Selection could therefore result in a spurious inference of population growth, but we believe it is far more likely that the rise in DENV-1 inferred from the genealogy reflects the actual rise in DENV-1 observed in the hospitalization data. In the future, however, it would be interesting to look at multistrain models that could encompass the competitive and facilitative interactions between different dengue genotypes and serotypes, as long as sufficient data are available.

We end by noting that the methods we used to estimate population dynamics and parameters from the DENV-1 genealogy could be greatly improved upon in the future. One of the shortcomings of the methods used here is that phylogenetic uncertainty in the genealogy is not fully taken into account. For DENV-1, the availability of whole genome sequence data meant that there was relatively little uncertainty in the genealogy and we showed our phylodynamic estimates were robust to this level of uncertainty. But in other cases where sequence data are less informative about the genealogy, phylogenetic uncertainty will need to be considered. Another shortcoming is that we only fit deterministic epidemiological models. Although methods exist for fitting stochastic models to genealogies, these methods assume that the population is unstructured such that all lineages can be assumed to be in the same population, and therefore cannot yet be applied to the type of structured coalescent models used here (Rasmussen et al. 2011). For dengue in southern Vietnam, stochasticity can reasonably be ignored because the large number of infections and the strong seasonal dynamics ensure that dynamics are unlikely to widely differ from what is expected under deterministic models. Yet, in other cases, stochasticity can play an important dynamical role, like at the beginning of epidemics when prevalence is low. As we have shown, fitting mechanistic models to genealogies can improve our understanding of the forces shaping genealogies and improve phylodynamic estimates; extending current methods to include phylogenetic uncertainty and stochasticity will help to further improve the robustness of phylodynamic inference.

## Materials and Methods

### Epidemiological Data

Dengue hospital admission data were compiled from the Hospital for Tropical Diseases and Children’s Hospitals 1 and 2 in HCMC, as described in Anders et al. (2011). We report the absolute number of dengue hospital admissions occurring each month. RT-PCR data on relative serotype frequencies is from Vu et al. (2010).

### Sequence Data and Tree Reconstruction

Whole-genome viral sequences were obtained through the Broad Institute’s Genome Resources in Dengue (GRID) website (www.broadinstitute.org/annotation/viral/Dengue/Home.html, last accessed October 29, 2013). For our analysis, 237 sequences were randomly subsampled from the larger set of 757 sequences used in the analysis of Vu et al. (2010). This larger set of sequences contained many samples collected during the same dengue season. We therefore subsampled sequences in years where large numbers of samples were sequenced so that approximately 40 sequences were included from each year between 2003 and 2008. Including more sequences did not appear to have any substantial effect on the population dynamics inferred from the genealogy. For each sequence we provide the Broad Institute’s ID, the GenBank accession number, the date of isolation and whether the sample was isolated from an individual identified as living in HCMC in supplementary table S1, Supplementary Material online.

The DENV-1 genealogy was inferred using the Bayesian MCMC methods available in BEAST version 1.6.1 (Drummond and Rambaut 2007). Phylogenetic inference was performed using a General Time Reversible substitution model with gamma rate heterogeneity across sites and a strict molecular clock across lineages. Coalescent times inferred under the strict molecular clock were very close to those inferred under a relaxed clock. A Bayesian Skyline prior was chosen as the tree prior with 20 different population size intervals (Drummond et al. 2005). Including more population size intervals did not substantially change the BSPs.

### Phylodynamic Inference

For each of the three mechanistic models considered, we were interested in estimating the posterior density of parameters *θ* and latent state variables 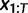 given the fixed DENV-1 genealogy 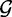. The variables in 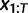 track the state of the population, such as the number of susceptible and infected individuals in the population. We can compute the trajectory of all state variables in 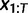 given a particular set of parameters *θ* by forward simulating the population dynamics from the deterministic ordinary differential equations (ODEs) that define the epidemiological model. For efficiency, forward simulations were performed using the Euler method of numerical integration with a sufficiently small integration time step.

Given a particular parameter set *θ* and population state trajectory 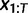, we need to be able to compute the likelihood of the coalescent model given the genealogy to compute the posterior probability of *θ* and 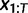. Methods for computing this likelihood for generic state space models were described in Rasmussen et al. (2011). For all of the coalescent models we consider here, the likelihood can be computed using an exponential probability distribution with rate parameter *λ*, the expected coalescent rate, which we derive for each model below.

A Metropolis–Hastings algorithm was used to sample from the posterior density of *θ* and 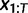. For each iteration, new parameters were proposed and either accepted or rejected based on the posterior probability of the parameters and the state trajectory simulated under the model. Uniform priors were placed on all parameters. The algorithm was tested on multiple genealogies simulated under each model before being applied to the DENV-1 genealogy. The algorithm was implemented in the program EpiTreeFit and Java source code is available from the project website (http://code.google.com/p/epitreefit/, last accessed October 29, 2013).

Bayes factors were used to compare the fit of different models to the DENV-1 genealogy. Bayes factors give the ratio of posterior to prior odds favoring one model over another and thus serve as a summary of the evidence provided by the data in favor of a given model (Kass and Raftery 1995). To compute Bayes factors from the MCMC samples, we used the standard harmonic mean estimator, which takes the harmonic mean of the posterior probabilities of the MCMC samples. Although the harmonic mean estimator is known to be unstable when MCMC methods are used to integrate over a very high-dimensional or complex parameter space (Lartillot and Philippe 2006), we found that Bayes factors computed from different MCMC runs were quite stable, with variances less than one.

### Epidemiological and Coalescent Models

Below, we describe the three epidemiological models we fit to the DENV-1 genealogy and show how the corresponding coalescent model for each of these model can be derived using the coalescent framework of Volz et al. (2009) and Volz (2012).

### Seasonal SIR Model

The first model we consider is a simple, unstructured SIR model with direct transmission between humans for a single dengue serotype. By considering DENV-1 dynamics in the absence of the other DENV serotypes, we are assuming that susceptibility to and infectivity with DENV-1 is not, or is only weakly, impacted by the other DENV serotypes over this time period. The model is given by the following system of ODEs:
(1*a*)
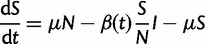

(1*b*)
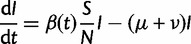

(1*c*)
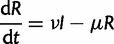

where *μ* is the human birth and death rate, *ν* is the recovery rate in humans, and 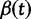 is the seasonally varying transmission rate. This transmission rate is given by the following equation:
(2)
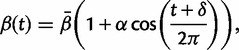

where 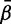 is the average transmission rate over the entire year, α is the seasonal amplitude parameter, and δ controls the seasonal phase. *R*_0_ in this model is given by 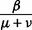.

To reduce the number of parameters in the model that need to be estimated directly from the genealogy, we fixed several parameters available from other demographic or clinical data. We fixed the human birth/death rate *μ* at 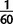 per year, reflecting the current birth rate in Vietnam, and the human population size at 10 million to reflect the population of HCMC, which was officially 7.5 million in 2007 but likely much larger (General Statistics Office of Vietnam 2008). The recovery rate *ν* was set at 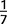 per day, consistent with observed durations of viremia between 2 and 12 days (Gubler et al. 1981; Tricou et al. 2011). The free parameters in the model that we estimated were the average transmission rate 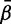, the seasonal amplitude *α*, the seasonal phase *δ*, and the initial conditions for the number of susceptible and infected individuals in the population.

As shown in Volz (2012), the pairwise rate of coalescence *λ* for an unstructured SIR model depends on the transmission rate, as well as the number of infected individuals and the fraction of the population susceptible to infection:
(3)
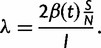



### Vector-Borne Model

Our vector-borne transmission model for an unstructured human population is given by the following ODEs:
(4*a*)
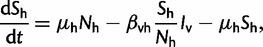

(4*b*)
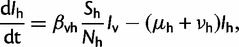

(4*c*)
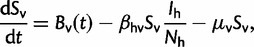

(4*d*)
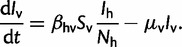

The subscripts “h” and “v” denote variables and parameters for humans and vectors, respectively. *B*_v_ is the vector birth rate, which we assume varies seasonally. The force of infection to both humans and mosquitoes is frequency-dependent with respect to humans. The transmission rates 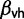 and 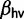 are proportional to the per capita biting rate of a mosquito times a factor that determines the probability of a bite being infectious. For this model, 
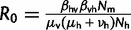
, as shown in Keeling and Rohani (2008).

We varied the size of the mosquito population by sinusoidally forcing the vector birth rate *B*_v_:
(5)
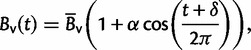

where 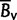 is the seasonal average of *B*_v_. We set 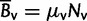, so that the average seasonal mosquito population size does not change over time. However, because we do not know the size of the mosquito population *N*_v_, we redefine 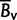 as equal to 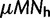, where *M* is a free parameter in the model that represents the ratio of the mosquito population size to the human population size.

When fitting the vector-borne model, we fixed the human birth and death rate 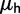, population size *N*_h_, and recovery rate 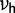 at the same values as in the directly transmitted model. We also fixed the vector death rate 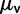 at 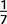 per day, which was chosen to represent the average of the daily mortality rates reported in the literature for *A. aegypti* adult females (Sheppard et al. 1969; McDonald 1977; Harrington et al. 2001). We initially allowed the transmission rates 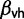 and 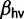 to differ depending on the directionality of transmission but model comparisons showed that a model with asymmetric transmission rates did not fit the genealogy significantly better than a model with symmetric rates (Bayes factor 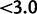). We therefore only estimated a single transmission rate, *β*. The other estimated parameters were seasonal amplitude *α*, the seasonal phase *δ*, the ratio of mosquitoes to humans *M*, and the initial conditions for the number of susceptible and infected humans in the population.

Given the forward-time dynamics, we need to derive the rate of coalescence under the vector-borne model. However, the population is now structured because viral lineages can either be in an infected human or an infected mosquito. We therefore use the structured coalescent framework of Volz (2012), who showed that for a generic structured population where lineages can be in any of *m* different states, the rate of coalescence 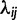 for two lineages *i* and *j* is
(6)
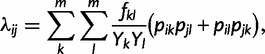

where *p_ik_* is the probability that lineage *i* is in state *k* and *p_jl_* is the probability that lineage *j* is in state *l*. *f_kl_* is the rate at which lineages are transmitted from state *k* to state *l* and *Y_k_* and *Y_l_* are the total number of infected individuals in states *k* and *l*, respectively.

Adapting equation (6) to the vector-borne model, the rate of coalescence becomes
(7)
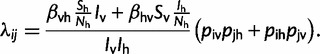

From equation (7), we can see that we need to compute the probabilities that lineages are in either an infected vector or human. We discuss how these lineage state probabilities can be computed in the supplementary appendix, Supplementary Material online.

### Spatially Structured Model

Our spatially structured model partitions the total population into two subpopulations, which we refer to as the focal and global populations. For our analysis of DENV-1, the focal population corresponds to HCMC and the global population to the non-HCMC population. The model is given by the following ODEs:
(8*a*)
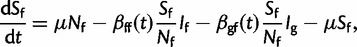

(8*b*)
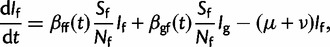

(8*c*)
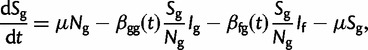

(8*d*)
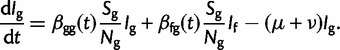

We assume that the human birth/death rate *μ* and recovery rate ν is the same in both populations, fixed at the values used for the previous two models. The global population size *N*_g_ was set at 25 million to reflect the population size of the southernmost 20 provinces excluding HCMC (General Statistics Office of Vietnam 2008).

Transmission between the two populations occurs when an infected individual from one population contacts a susceptible individual in the other population. Bayes factor comparisons revealed that a model with separate transmission rates 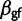 and 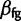 did not fit the DENV-1 genealogy significantly better than a model with a single between-population transmission rate 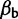 (Bayes factor 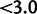). We therefore set 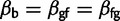. However, the transmission rates within the focal population 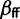 and global population 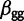 are allowed to differ.

We first fit a model with seasonality in the focal population using the same sinusoidal forcing function as in equation (2) and assuming no seasonality in the non-HCMC population. For this model, we estimated the transmission rates, 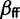, 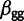, and 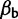, as well as the seasonality parameters *α* and *δ* for the focal population. We also estimated the initial number of susceptible and infected individuals in the focal population but, to reduce the number of parameters being fit, we set the initial conditions for the global population to their expected values at endemic equilibrium. We also fit a second model that allowed for seasonality in both populations. In this case, we estimated the seasonality parameters α and δ for both populations, as well as the initial conditions in the non-HCMC population. Although Bayes factors indicated that the more complex model with seasonality in both populations did not fit the genealogy significantly better, we retain this parameterization because it allowed us to detect the differing seasonal phase between populations.

We can again use the coalescent rate given in equation (6) to derive the coalescent rate for our spatially structured model. For two lineages *i* and *j* the pairwise rate of coalescence is as follows:
(9)
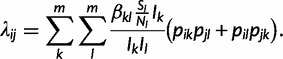

In this case, there are only two populations and the subscripts *k* and *l* refer either to the focal or the global population. Given our epidemiological model, the lineage state probabilities *p_ik_* can be computed backward in time using equation (42) in Volz (2012), given the state of each lineage at the time of sampling.

## Supplementary Material

Supplementary appendix, table S1, and figures S1–S6 are available at *Molecular Biology and Evolution* online (http://www.mbe.oxfordjournals.org/).

Supplementary Data
