# Supplementary material for: Reconciling Phylodynamics with Epidemiology: The Case of Dengue Virus in Southern Vietnam
Source: Mol Biol Evol. 2013 Oct 22;31(2):258–71. doi: 10.1093/molbev/mst203 (PMC3907054; doi:10.1093/molbev/mst203)
Supplement: Supplementary Data [file supp_31_2_258__index.html]

Reconciling phylodynamics with epidemiology: The case of dengue virus in southern Vietnam — Reconciling Phylodynamics with Epidemiology: The Case of Dengue Virus in Southern Vietnam — Reconciling Phylodynamics with Epidemiology: The Case of Dengue Virus in Southern Vietnam — Supplementary Data 

# Reconciling Phylodynamics with Epidemiology: The Case of Dengue Virus in Southern Vietnam

## Supplementary Data

files

**Files in this Data Supplement:**

- Supplementary Data - pdf file
- Supplementary Data - pdf file
- Supplementary Data - csv file
