# Supplementary material for: Reconciling Phylodynamics with Epidemiology: The Case of Dengue Virus in Southern Vietnam
Source: Mol Biol Evol. 2013 Oct 22;31(2):258–71. doi: 10.1093/molbev/mst203 (PMC3907054; doi:10.1093/molbev/mst203)
Supplement: Supplementary Data [file supp_mst203_RasmussenFinalMBESuppAppendix.pdf]

Supplementary Appendix

The pairwise rate of coalescence under our vector-borne epidemiological model is:

$$\lambda_{ij} = \frac{\beta_{vh}\frac{S_h}{N_h}I_v + \beta_{hv}S_v\frac{I_h}{N_h}}{I_v I_h}\left(p_{iv}p_{jh} + p_{ih}p_{jv}\right), \tag{1}$$

where p_{iv}, for example, gives the probability that lineage i is in a vector.

In general, we can compute the probability that a given lineage is in a certain state if we know the initial state of the lineage at the time of sampling and the rates at which lineages move between states. How the lineage state probabilities change as we move backwards in time can be tracked using master equations (Volz, 2012). For our vector-borne model, assuming that the number of infected humans and vectors is large relative to the number of lineages in the genealogy, the master equations for the vector and human states are:

$$\frac{dp_{iv}}{ds} = p_{ih}\frac{\beta_{vh}\frac{S_h}{N_h}I_v}{I_h} - p_{iv}\frac{\beta_{hv}S_v\frac{I_h}{N_h}}{I_v} \tag{2a}$$

$$\frac{dp_{ih}}{ds} = p_{iv}\frac{\beta_{hv}S_v\frac{I_h}{N_h}}{I_v} - p_{ih}\frac{\beta_{vh}\frac{S_h}{N_h}I_v}{I_h}. \tag{2b}$$

From these master equations, we can see that the rate at which probability mass flows between states depends on the rates at which lineages move between states through transmission events.

Here, we assume that the lineage state probabilities are at equilibrium with respect to the overall epidemiological dynamics. This is a reasonable assumption as long as the lineages move between states much faster than the overall epidemiological dynamics change. With this assumption, we can then solve for the equilibrium probabilities p^*_{iv} and p^*_{ih} using equation (2). To do so, we set $\frac{dp_{iv}}{ds} = 0$ and substitute in $1 - p_{iv}$ for p_{ih}. Solving, p^*_{iv} becomes:

$$p^*_{iv} = \frac{\frac{\beta_{vh}\frac{S_h}{N_h}I_v}{I_h}}{\left(\frac{\beta_{vh}\frac{S_h}{N_h}I_v}{I_h} + \frac{\beta_{hv}S_v\frac{I_h}{N_h}}{I_v}\right)}, \tag{3}$$

and

$$p_{ih}^{*} = 1 - p_{iv}^{*} = \frac{\frac{\beta_{hv} S_v \frac{I_h}{N_h}}{I_v}}{\left(\frac{\beta_{vh} \frac{S_h}{N_h} I_v}{I_h} + \frac{\beta_{hv} S_v \frac{I_h}{N_h}}{I_v}\right)}. \tag{4}$$

Plugging these equilibrium lineage state probabilities into (1), the pairwise rate of coalescence becomes:

$$\lambda_{ij} = \frac{\beta_{vh} \frac{S_h}{N_h} I_v + \beta_{hv} S_v \frac{I_h}{N_h}}{I_v I_h} \left(\frac{2 \frac{\beta_{vh} \frac{S_h}{N_h} I_v}{I_h} \frac{\beta_{hv} S_v \frac{I_h}{N_h}}{I_v}}{\left(\frac{\beta_{vh} \frac{S_h}{N_h} I_v}{I_h} + \frac{\beta_{hv} S_v \frac{I_h}{N_h}}{I_v}\right)^2} \right). \tag{5}$$

We can make sense of this coalescent rate by decomposing it into two parts. One part, the term in parentheses on the right hand side, gives the overall probability of the two lineages being in different states: one in an infected vector and one in an infected human. Intuitively, this term enters into the coalescent rate because coalescent events can only occur at transmission events, which requires the two lineages to be in opposite states. Because of this requirement, we can see that the rate of coalescence will generally be lower for a vector-borne pathogen than a directly transmitted pathogen. The probability that two lineages are in opposite states reaches a maximum when $p_{iv} = p_{ih} = \frac{1}{2}$, which means that the highest attainable probability of the lineages being in opposite states is also $\frac{1}{2}$. All else being equal then, the rate of coalescence for a vector-borne pathogen will be at most half that of a directly transmitted pathogen.

The other part of the coalescent rate, the leading term on the right hand side of (5), gives the rate at which two lineages coalesce conditional on one lineage being in a vector and the other in a human. We can see that the coalescent rate inversely depends on the product of the number of infected vectors and humans, as this gives the probability that of all the lineages circulating in the population, the pair of lineages that we are considering are the two lineages that coalesce at a given transmission event. Because the term $I_v I_h$ tends to dominate the overall rate of coalescence, the number of infected humans and vectors plays

a very important role in determining the overall coalescent rate. The number of infected humans and vectors in turn depends on a key parameter M, which we define as the ratio of the vector population size N_v to the human population size N_h.

To understand how the vector population size N_v affects the rate of coalescence, we can hold R_0 constant, so that the number of infected humans remains the same at equilibrium, but vary the ratio of vector to human population sizes M (note that we decrease the transmission rates β_{vh} and β_{hv} as we increase M to keep R_0 constant). At equilibrium, the rate of coalescence for the vector-borne pathogen drops off asymptotically with increasing M relative to a directly transmitted pathogen (Supplementary Figure 4A). This is because the number of infected vectors increases with M (Supplementary Figure 4B), resulting in a larger product $I_v I_h$ in the denominator of (5), and consequently a lower coalescent rate.

References

Volz EM. 2012. Complex population dynamics and the coalescent under neutrality. *Genetics.* 190:187–201.
