# Supplementary material for: Reconciling Phylodynamics with Epidemiology: The Case of Dengue Virus in Southern Vietnam
Source: Mol Biol Evol. 2013 Oct 22;31(2):258–71. doi: 10.1093/molbev/mst203 (PMC3907054; doi:10.1093/molbev/mst203)
Supplement: Supplementary Data [file supp_mst203_RasmussenFinalMBESuppFigures.pdf]

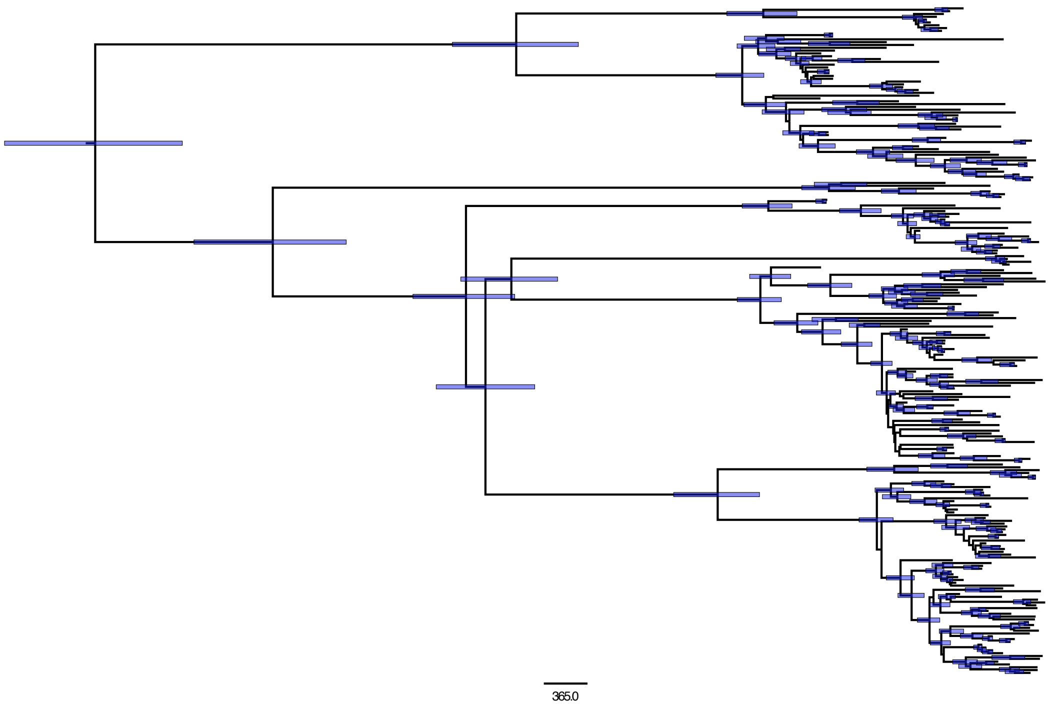

Supplementary Figure 1: Maximum clade credibility tree for DENV-1. The 95% credible intervals on the coalescent times are shown as blue bars. The scale bar shows time in days.

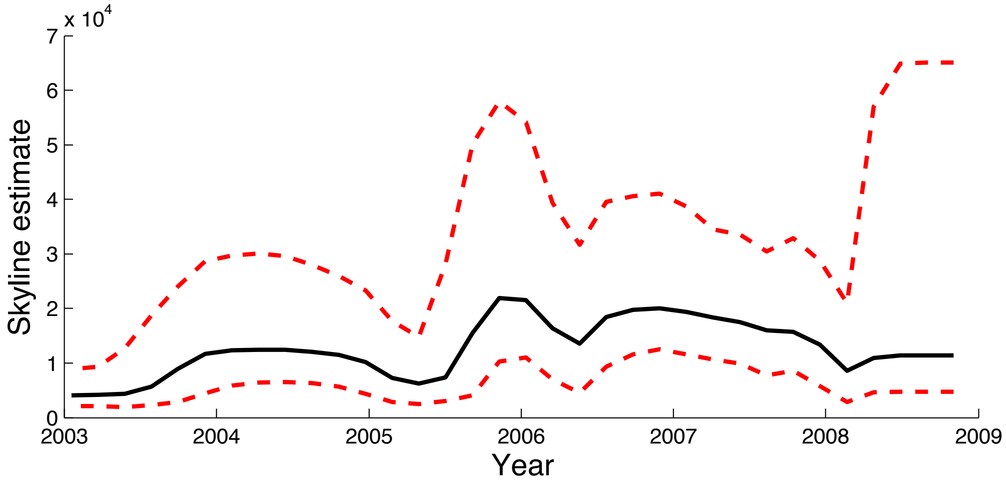

Supplementary Figure 2: Bayesian Skyline Plot inferred from the HCMC DENV-1 sequences with all non-HCMC sequences removed. Black lines show the median posterior estimates and dashed red lines give the 95% credible intervals.

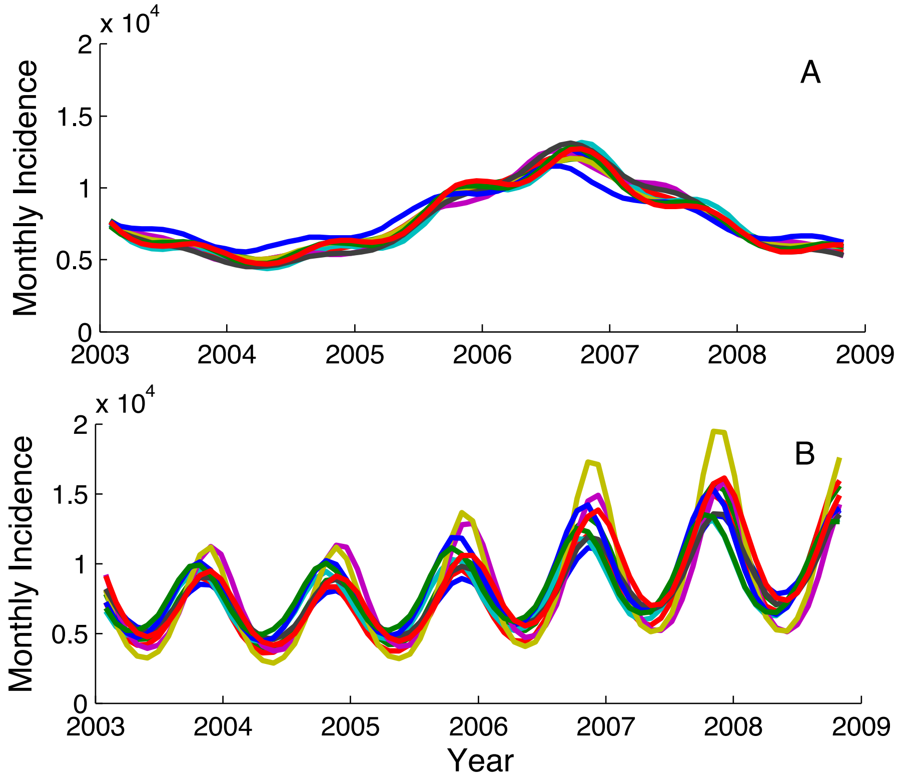

Supplementary Figure 3: Reconstructed incidence of DENV-1 inferred from ten genealogies randomly sampled from the posterior distribution of trees. Only the median estimate from each tree is shown. (A) Estimates under the unstructured seasonal SIR model. (B) Estimates under the combined model with both spatial structure and vector-borne transmission.

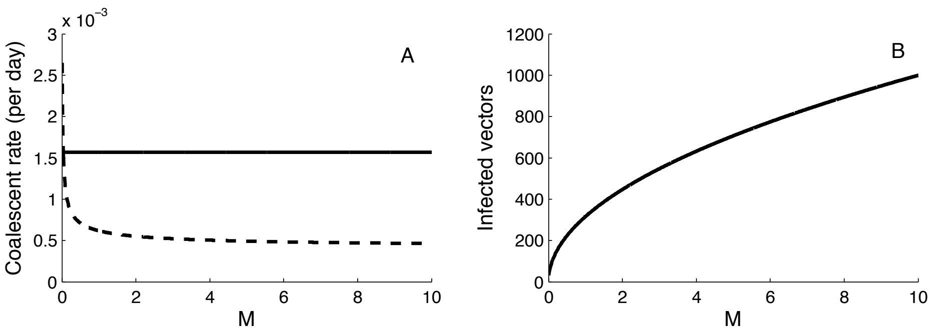

Supplementary Figure 4: (A) Comparison of coalescent rates at equilibrium for the direct transmission model (solid) and for the vector-borne model (dashed) over a range of M. For the direct transmission model, the coalescent rate does not depend on M. (B) The number of infected mosquitoes at equilibrium under different values of M. The number of infected humans remains constant regardless of M because we are holding R_0 constant.

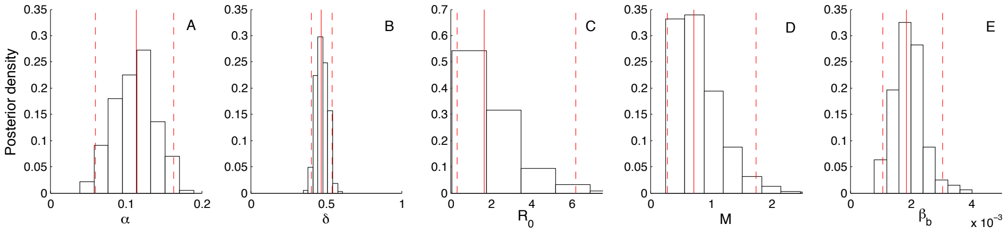

Supplementary Figure 5: Posterior densities of the parameters inferred from the DENV-1 genealogy under the combined model with both spatial structure and vector-borne transmission. (A) The seasonal amplitude α in HCMC. (B) The seasonal phase parameter δ for HCMC. (C) The basic reproduction number R_0. (D) The ratio of mosquito to human population sizes M in HCMC. (E) The transmission rate β_b between HCMC and the non-HCMC provinces.

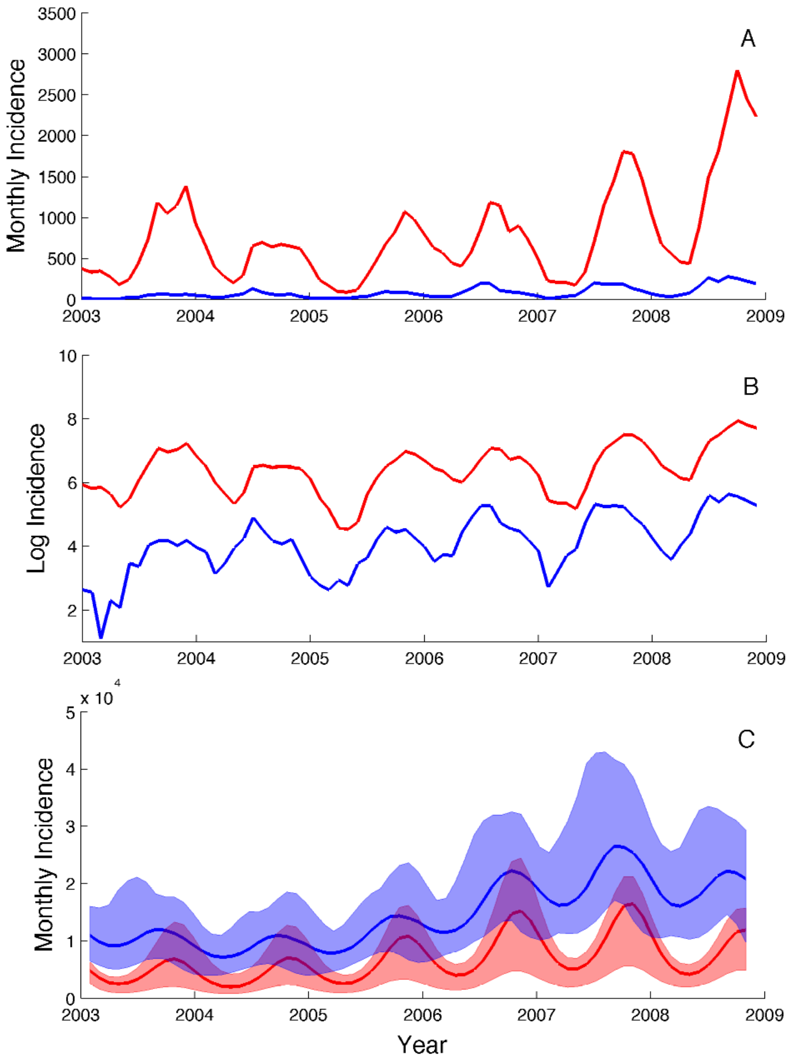

Supplementary Figure 6: Population dynamics of dengue in HCMC (red) and the non-HCMC provinces (blue). (A) Monthly dengue hospital admissions in HCMC by location of patients' primary place of residence. The small number of cases from the provinces likely reflects the low probability of dengue patients in the provinces being hospitalized in HCMC. (B) Same as in A but on a log scale to emphasize the difference in seasonal phase between HCMC and the provinces. (C) Incidence inferred under the spatially structured model for the HCMC and non-HCMC populations. Shaded regions give the 95% credible intervals.
